# Weaning from long-term mechanical ventilation utilizing closed-loop ventilation mode (IntelliVent^®^-ASV^®^) in a patient with spinal cord injury

**DOI:** 10.1038/s41394-018-0082-7

**Published:** 2018-06-19

**Authors:** Satoru Shimizu, Masashi Nakajima, Masayuki Yamazaki, Takashi Nagayama, Ryuta Suzuki

**Affiliations:** Ward for Patients with Neurological Disabilities, Tsurumaki-onsen Hospital, 1-16-1 Tsurumaki-kita, Hadano-shi, Kanagawa 257-0001 Japan

## Abstract

**Introduction:**

Cervical spinal cord injury with the C3 neurological level may cause respiratory failure and require long-term mechanical ventilation. Conventional weaning of spontaneous breathing trials is difficult to perform outside of intensive care or spinal cord units.

**Case presentation:**

An 80-year-old man presented with total tetraplegia and restrictive respiratory failure that required assisted ventilation after a falling accident. Cervical spine magnetic resonance imaging showed cervical cord compression that was worst at the C3–C4 intervertebral level. He experienced unexpected cardiac arrest during the conventional weaning process of trials of intermittent spontaneous breathing in the intensive care unit. The automated weaning protocol utilizing a closed-loop ventilation mode (IntelliVent^®^-ASV^**®**^) was introduced 131 days after injury in our ward for chronically ill patients. The patient was successfully weaned 39 days after the introduction of the weaning protocol.

**Discussion:**

An automated weaning protocol utilizing a closed-loop ventilation mode could be an optional procedure in patients with cervical cord injury on long-term mechanical ventilation, even in a ward for chronically ill patients where sufficient staff is not available. The efficacy and safety, and the cost-effectiveness of the procedure should be examined in larger spinal cord units.

## Introduction

Cervical spinal cord injury with the C3 neurological level, according to the International Standards for the Neurological Classification of Spinal Cord Injury (ISNCSCI), may cause partial denervation of the diaphragm, paralysis of the thoracic and abdominal walls, and require mechanical ventilation [1]. Weaning from mechanical ventilation in these patients may take a longer time and be exertive compared to patients with acute respiratory failure in intensive care units (ICU). In acute care settings, closed-loop control ventilation has been used since the 1990s, which facilitates automated weaning from the ventilator that is safe and efficient in terms of the workload for the health care team [2]. We hypothesized that this automated weaning would be applicable to a patient with long-standing respiratory failure due to cervical cord compression.

## Case presentation

An 80-year-old man with known cervical canal stenosis due to ossification of the cervical posterior longitudinal ligament was transferred to an emergency room soon after a falling accident. The patient was alert and had complete tetraplegia, sensory deficits below the C4 cervical sensory level, and progressive restrictive respiratory failure that required assisted ventilation, and, eventually, a tracheostomy. Cervical spine magnetic resonance imaging showed cervical cord compression that was worse at the C3–C4 intervertebral level, and an intramedullary high signal at C3 and C4 vertebral levels, and the patient was managed conservatively. During the conventional weaning process of trials of intermittent spontaneous breathing in the ICU, an unexpected cardiac arrest made both the patient and medical staff reluctant to proceed with further weaning. Fifty-eight days after cervical cord injury, the patient was transferred to our hospital for further rehabilitation. He was fully conscious and received pressure-support mechanical ventilation with back-up rates of 16 breaths. He had total tetraplegia below shoulder girdle muscles with a preserved sensation from C2 to C3 on both sides, and a urinary obstruction. The limbs and trunk were stiff with contracture, and muscle stretch reflexes of the limbs were abolished. Neurological level of injury was C3 according to the ISNCSCI, with a total sensory score of four. There was no sensory sacral sparing, while the deep anal pressure was preserved, and the American Spinal Injury Association scale graded B. Chest roentgenogram and computerized tomography showed bilateral, widespread atelectasis. The patient occasionally showed signs of pulmonary infection. Intensive pulmonary care and rehabilitation, including physical support for expectoration, mechanically assisted removal of tracheal secretions (CoughAssist E70^®^, Philips Japan, Tokyo), ventilator muscle training, and management of the spasticity of the abdominal wall, resulted in the improvement of the roentgenological findings. His expiratory tidal volume (*V*_T_) measured 150 mL.

Although the neurological status according to the ISNCSCI was unchanged, the patient was willing to be weaned from mechanical ventilation as his general condition improved. After informed consent was given, we attempted automated weaning from mechanical ventilation using IntelliVent^®^-ASV^**®**^ on and after 131 days of injury. IntelliVent^®^-ASV^**®**^ (Hamilton Medical AG, Switzerland) is a closed-loop ventilation mode that adjusts the pressure support in terms of the percentage of the ideal minute volume (%MV). The ideal MV (100%MV) is calculated from the patient’s height and gender. Based on Otis’ equation [3], adaptive support ventilation will select the best *V*_T_–respiratory rate (RR) coupling for the optimal work of breathing. IntelliVent^®^-ASV^**®**^ has interrelated functions: an auto-adjustment for carbon dioxide (CO_2_) elimination, an auto-adjustment for oxygenation, an auto-weaning tool named Quick Wean (QW), and a spontaneous breathing trial (SBT). The setting of the target %MV is automated based on either the monitored end-tidal CO_2_ or monitored spontaneous breathing rate. The auto adjustment of fraction of inspiratory oxygen or positive end-expiratory pressure is based on the monitored oxygen saturation fraction of hemoglobin with a pulse oximeter (SpO_2_) [4]. The QW mode is an optional automated weaning, and progressively reduces the pressure support, monitors for readiness-to-wean criteria, and provides the option to automatically conduct a fully controlled SBT. In the QW mode, when the spontaneous rate is less than the upper limit of the predicted target range, %MV is automatically decreased gradually to the selected level. To facilitate the recovery of *V*_T_ by means of the loading work on respiratory muscles, we set the level of support to 70%MV for the QW mode. When spontaneous breathing satisfied the pre-determined conditions of oxygenation and ventilation for 1 min, the SBT mode was used. We operated the SBT mode during the day from 9:00 a.m. to 6:00 p.m.

**Fig. 1:**
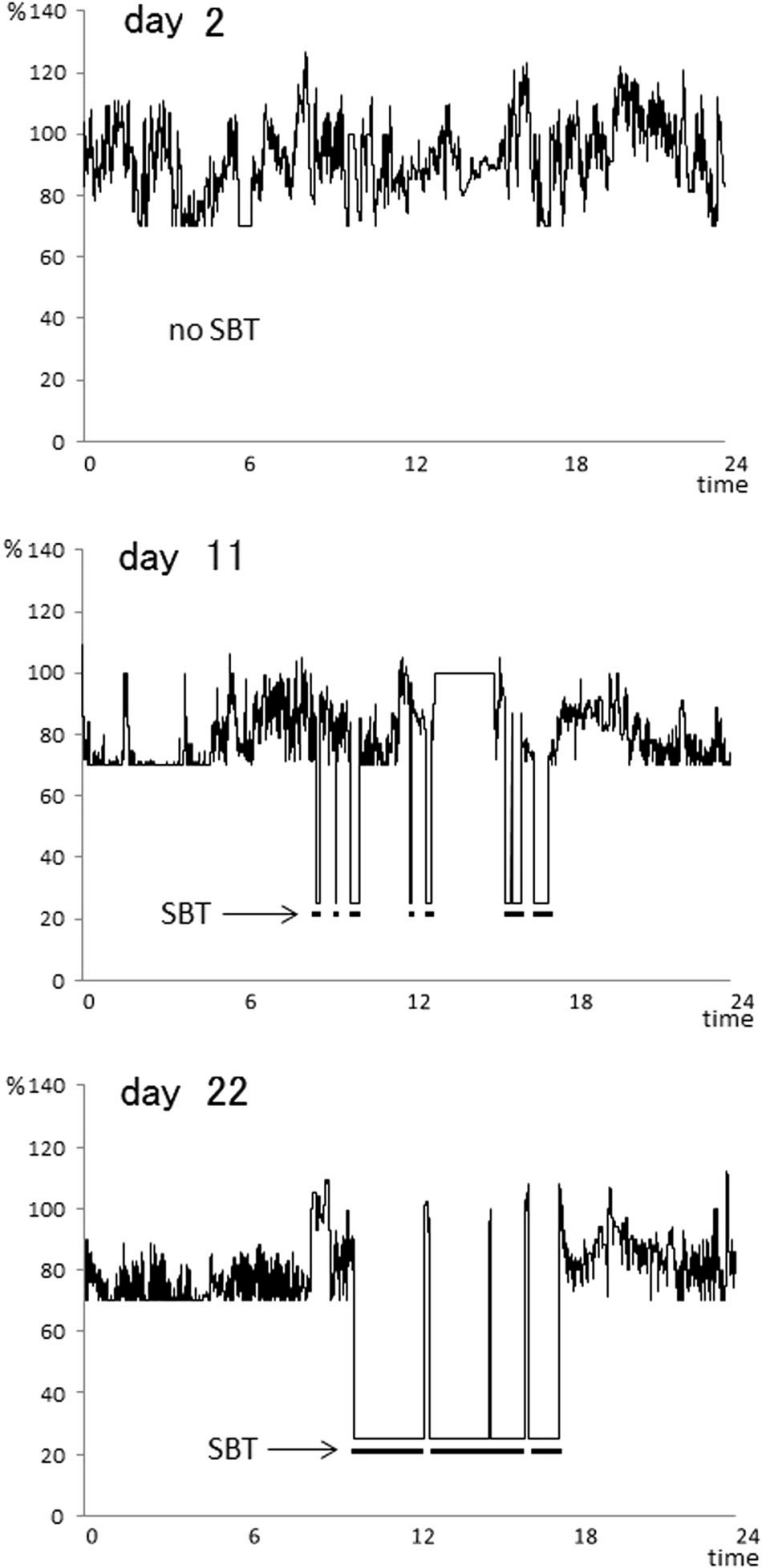
A gradual increase of the duration of the QW and SBT modes. On day 2, after the operation of the QW mode, 70%MV was seldom achieved (upper row). On day 11, the QW (70%MV) time increased, and the SBT (25% MV) time appeared intermittently (middle row). On day 22, the QW time further increased, and SBT ran almost fully during the daytime (lower row)

**Fig. 2:**
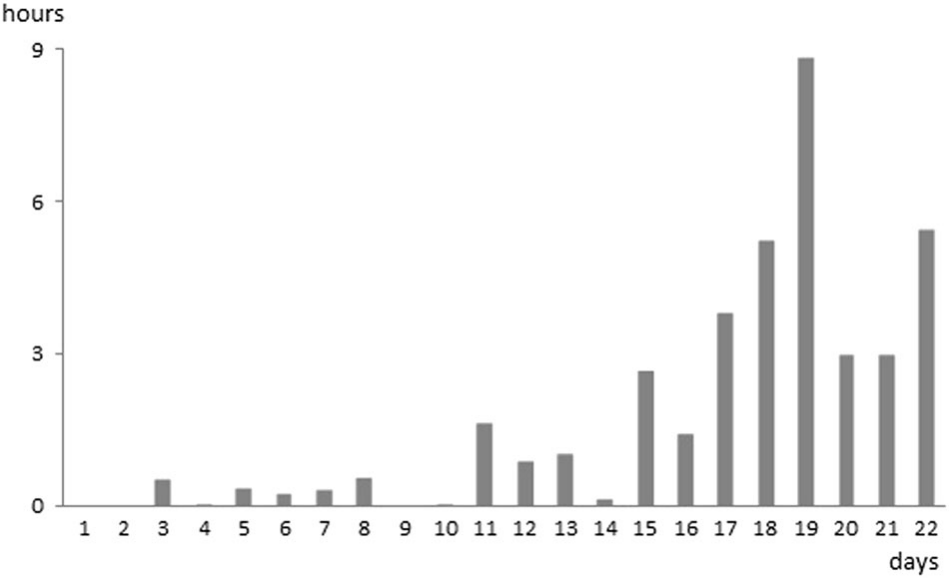
Progressive increase of the SBT time during an automated process of weaning with the IntelliVent^®^-ASV^®^. The number on the *x*-axis indicates the days after the introduction of automated weaning. The ordinate indicates the duration of SBT in hours

In the QW mode, the duration of 70%MV and SBT time (25%MV) increased gradually (Figs. 1 and 2). Twenty-two days after the introduction of the QW and SBT modes in association with intensive pulmonary rehabilitation, SBT was running almost fully during the daytime. At this time, the expiratory *V*_T_ was increased to 350 mL from 150 mL upon admission to our hospital. Thereafter, we withdrew the pressure support ventilation during the nighttime 39 days after the introduction of the auto-weaning mode, and the patient’s weaning process was completed.

## Discussion

Respiratory complications are the leading cause of morbidity and mortality in the short and long term after cervical spinal cord injury [5]. Most of the patients with a complete injury at or above the C5 cord level will require mechanical ventilation and a tracheostomy. A substantial number of these patients will recover spontaneous ventilation after following an adequate protocol of weaning from the respirator [6]. The weaning process conventionally uses SBT, which gradually increases the spontaneous breathing time manually. This process, however, takes place in the ICU or in the spinal cord unit [5, 6]. The medical staff in our ward is composed of two neurologists and one internist. A medical engineer who has expertise on ventilator machines offers round-the-clock care with one of the medical staff. The ratio of nurses/beds is 1:10. In such circumstances, an ordinary weaning process that requires frequent observations of patients during SBT is elaborative.

This case report showed safe ventilator weaning using an automated weaning program combined with the closed-loop ventilation mode. IntelliVent closed-loop control ensures that the patient is never apneic, does not have too large or too low *V*_T_, or too high or too low RR [4]. Our patient had an extended injury, mainly at the C4 spinal cord level, and may have had partial denervation of the diaphragm and paralysis of the intercostal muscles. The patient was preoptimized by means of pulmonary care and rehabilitation before the weaning process [7]. After respiratory optimization, his *V*_T_ of 150 mL suggested an incomplete paralysis of the diaphragm and the potential to be weaned from mechanical ventilation [5, 6]. After a long-standing dependency on the ventilator for 4 months, he was successfully weaned 39 days after the introduction of an automated weaning tool. The length of the weaning process is comparable to those reported in a case series of conventional weaning for spinal cord-injured patients in the settings of an intermediate respiratory care unit [5] and rehabilitation clinic [6]. The recovery of our patient’s expiratory *V*_T_ from 150 to 350 mL after the auto-weaning process may be due to reinnervation of the diaphragm, stabilization of the chest wall, and reduction of abdominal compliance [1]. Accompanied by pulmonary care and rehabilitation, an automated weaning protocol utilizing the closed-loop ventilation mode could be an optional procedure in patients with a cervical cord injury on long-term mechanical ventilation, even in a ward in which sufficient monitoring staff is not available. The efficacy and safety, and the cost-effectiveness of the procedure should be examined in larger spinal cord units.
